# The Effects of Urban Living Conditions on Subjective Well-Being: The Case of German Foreign Service Employees

**DOI:** 10.1007/s11482-023-10169-w

**Published:** 2023-04-22

**Authors:** Heiko Rüger, Stefanie Hoherz, Norbert F. Schneider, Herbert Fliege, Maria M. Bellinger, Brenton M. Wiernik

**Affiliations:** 1 Federal Institute for Population Research (BiB), Friedrich-Ebert-Allee 4, 65185 Wiesbaden, Germany; 2 Federal Foreign Office, Berlin, Germany; 3 Department of Psychology, University of South Florida, Tampa, US

**Keywords:** Subjective well-being, Urban environmental and living conditions, Expatriates, Megacity, Dominance analysis, Human development index

## Abstract

In an increasingly urbanized world, understanding the determinants of urban well-being will continue to grow in importance. Although the effects of different indicators of living conditions on well-being have been widely studied individually, little is known about their relative impact when examined jointly. In this study, we use a unique multi-source dataset that allows us to investigate the effect and relative importance of a variety of subjectively and objectively assessed aspects of urban living conditions on the subjective well-being (SWB) of German Foreign Service expatriates. The study captures living conditions in metropolises around the world at different stages of development, and assesses living conditions in a culturally comparably homogeneous set of participants, thus being potentially less confounded with cultural differences. Using linear regression and dominance analysis, we find that ‘quality of and access to nature’ (i.e., green space), ‘quality of housing’, and ‘quality of public goods’ (i.e., water, air, and sewage systems) have the strongest associations with SWB. Subjectively rated characteristics show stronger associations with SWB than externally assessed characteristics. Additionally, we examine whether the size of a city or the level of development of a country has an effect on SWB. Both living in a megacity (≥ 10 million inhabitants) and a lower development status have negative effects on SWB. However, these effects disappear when the various indicators of living conditions are controlled for. Our findings can inform organisations sending employees abroad as well as urban planners seeking to improve their policies and decision-making.

**Supplementary Information:**

The online version contains supplementary material available at 10.1007/s11482-023-10169-w.

## Introduction

The impact of urban living conditions on subjective well-being (SWB) is of growing interest for both researchers and policy makers (e.g., Ettema & Schekkerman 2016; Weckroth et al., 2022). Studies have investigated the impact of a variety of specific environmental factors, ranging from ecological problems to public transport, on well-being (e.g., Aretz et al., 2019; Levinson, 2020). The empirical evidence for a number of indicators of living conditions’ impact on SWB is fairly strong.

However, we know little about their relative impact — which indicators of living conditions contribute most to individuals’ well-being and which contribute, perhaps, less when compared to others? We also know very little regarding whether various indicators of urban living conditions interact and jointly impact well-being.

The primary aim of this study is therefore to investigate the relative importance of diverse aspects and measures of urban living conditions for SWB, by using multi-source data collected from a sample of German Foreign Service (GFS) expatriates. For this assessment, eleven well-established dimensions of environmental and other living conditions are included: ‘safety’, ‘quality of and access to nature’, ‘noise’, ‘housing’, ‘contact’, ‘congestion’, ‘quality of water, air, food supply, and sewage system’, ‘public transport/transit’, ‘accessibility of airport’, ‘sports opportunities’, and ‘medical care’.^1^ In addition to the subjective data from the GFS expatriates, this study integrates comparable information on more objectively assessed living conditions by a third party, i.e., Mercer.

Mercer is one of the world’s largest private-sector management consultancies, advising companies and organizations on, among other things, foreign assignments (Mercer, 2022a). Mercer regularly conducts a worldwide comparative study to evaluate the quality of life of expatriates in more than 500 cities (most recent survey from 2019) (Mercer, 2022b). These data are intended to help governments and major companies place employees on international assignments. According to the company’s own information, Mercer collects data from various sources, e.g. the WHO, the Centers for Disease Control, the Overseas Security Advisors Council, and others. Mercer includes evaluations of field researchers and consultants, who work in their network of offices worldwide, and it performs internal consistency and progress checks, and compares them with various standards. Mercer’s data on cities’ quality of life are considered by researchers to be objective data which can also be used for benchmarking (Morais et al., 2013).

By using this kind of objective information we make a contribution to the literature incorporating both data sources and comparing the subjective and objective assessments of living conditions (e.g., Ettema & Schekkerman 2016). Some authors have described this approach as ideal for creating a reliable picture of the impact of living conditions (e.g., McCrea et al., 2006). So far, few studies have combined the two types of indicators due to their differing nature and low availability. We will investigate what these objective measures can contribute to explaining SWB beyond their subjective counterparts, as well as how both types of indicators are related.

Research on the effects of living conditions usually surveys residents of one certain area or city (for an exception, see Leyden et al., 2011). However, research that focuses on only one selected metropolitan area or city is not representative in terms of location. In this respect we aim to go further and capture the living conditions of metropolises all over the globe and across different levels of development, using data from the German Federal Foreign Office’s about 230 diplomatic missions abroad (a total of 2,250 employees participated in the survey). The study presented here includes cities in more than 150 countries around the world (with 53 cities identifiable by name). While the selection of cities is not random, the study is far more representative than previous studies in terms of the number and geographic coverage of cities. German diplomatic missions are found in almost all government capitals worldwide, as well as in many other cities around the globe that are politically, economically, or culturally significant for international relations. Thus, our data should give a more universal picture of how individual SWB is associated with different aspects of urban settings. Studies with self-reported SWB across different countries also have the drawback that assessment is biased by (statistically uncontrollable) cultural differences (Veenhoven, 2012). This should be less of a problem for this study, as the assessment of living conditions was made by expatriates of the German Foreign Service who are a more culturally homogeneous group than random city dwellers across the world. Even though our dataset is clearly not representative of the broader population of the cities in question, this has some advantages. For instance, the relatively short time that expatriates spend in a location means habituation is less likely to blur their assessments of living conditions and potentially mitigate negative effects. Furthermore, expatriates are a particularly interesting study population as globalisation has led to an increasing number of employees facing the challenge of working and living abroad for a period of time (Takeuchi & Chen, 2013). The well-being of this group is therefore worthy of closer attention. So far it has not been studied specifically in surveys on living conditions.

Additionally, the available data allow us to carry out separate analyses to examine whether the size of a city or the level of development has an effect on SWB. These are pertinent questions as the global population is increasingly concentrated in cities many or most of which are rapidly growing in size. Indeed, since the 1990s, the overall population of cities with at least half a million people has doubled, while that of megacities has increased by more than three and a half times (United Nations, 2019). Almost 25% of the world’s population is currently living in cities with more than one million inhabitants and 8.4% are living in one of the world’s 35 megacities with more than 10 million inhabitants (Demographia, 2021). Consequently, risk factors such as pollution, overcrowding, housing issues, crime, noise and air pollution may be increasing (Peen et al., 2010), especially in low- to lower middle-income countries (United Nations, 2019). Previous research is mostly limited to analysing urban settings in the developed world, usually due to greater data availability. This makes it necessary to understand more about how living conditions affect SWB in various contexts, such as megacities or less developed countries.

The aim of the study is to examine the SWB and urban quality of life of German Foreign Service employees. This is not a representative sample of city residents, but one with a comparatively secure occupational position, stable income and good opportunities to live in better urban residential locations on postings abroad.^2^ Whether there are specific dimensions of quality of life that are particularly relevant for a sample with these characteristics has not yet been clarified. To our knowledge, studies of Foreign Service personnel and their attitudes toward environmental conditions are not available. Studies of other groups of people suggest that the importance of various living and environmental conditions, and their relationship to life satisfaction and well-being may differ by certain characteristics such as education and income.

The question of how important environmental factors are perceived by individuals is not independent of their knowledge, attitudes and concerns about the environment. Environmental knowledge enables the individual to more accurately assess the risk of pollution and thus increases their attention to environmental issues (Cheng et al., 2022; Li et al., 2014). For example, in a study of the effects of air quality on life satisfaction, it was found that the life satisfaction of environmentally aware people was more affected by air pollution than others (Luechinger, 2009).

While there is evidence that people with higher levels of education or income express more concern about their environment (Xiao & McCright, 2007; Meyer et al., 2022), there is other evidence from the German Socio-Economic Panel 1994–2019 showing that previously important group differences in the German population with respect to environmental concerns have increasingly levelled out in recent years (Hartmann & Preisendörfer, 2021). Evidence on air pollution, for example, suggests that residents of richer residential areas are more sensitive to air pollution than residents of lower-quality neighbourhoods (Cheng et al., 2022). On the other hand, however, according to some studies, it is people with a lower education who, for example, benefit to a greater extent from the presence of green spaces in their residential environment (Ambrey & Fleming, 2014) and frequent green spaces more often than people with higher education (Peschardt et al., 2012). In addition, higher earners are found to be more likely to protect themselves from the negative effects of air pollution, for example, through air filters (Zhang et al., 2017).

Overall, although the findings indicate that the importance of quality-of-life criteria of the living environment might differ for different socioeconomic groups, the empirical results so far do not yet allow for clear hypotheses.

In summary, this study uses a unique dataset which enables us to examine the effect of a large variety of different subjective *and* objective indicators of living conditions on SWB. We are particularly interested in the relative impact of these living conditions.

## Theoretical Background

According to Oishi and colleagues (2018), the term ‘subjective well-being’ (SWB) refers to the level of well-being people experience based on their subjective evaluations of their lives. Studies that merely focus on objective environmental indicators do not take the importance of such subjective evaluations into account. Some studies investigate a direct link between objective living conditions and well-being on one hand, but additionally include subjective characteristics as potential factors (e.g., Ettema & Schekkerman 2016; Weckroth et al., 2022). Others conceptualise the relationship between objective living conditions and SWB indirectly by including subjective evaluations of these conditions (McCrea et al., 2006). There are two main approaches to how this relationship between perceived living conditions and SWB can be conceptualised, and both approaches present arguments that we adopt in this study.

First, so-called *top-down models* assume that satisfaction with different life domains is the result of an overall satisfaction with life, and that this evaluation is largely driven by factors lying in the person (Headey & Wearing, 1989). Living conditions represent one of several life domains. According to this model, the evaluation of living conditions depends highly on how they are perceived by the individual and, presumably, to a lesser degree on the actual measures of, for example, water, air or noise pollution. For this reason, subjective evaluations of living conditions are central to this study. Furthermore, SWB has been shown to be associated with, among other things, personality traits such as neuroticism and extraversion (e.g., DeNeve & Cooper 1998). They thus perhaps do not just influence the attribution and subjective report process of SWB, but rather of living conditions as well. In order to get a better understanding of the degree of ‘subjectivity’ and the robustness of the measures, it is important to consider the role of personality traits (Lucas, 2018).

Second, so-called *bottom-up models* in quality-of-life research (e.g., Marans & Rodgers 1975; Diener, 1984) assume that overall SWB is a result of satisfaction with different life domains, including, for example, family life, work life, or satisfaction with the living surroundings (Sirgy & Cornwell, 2002; Cao, 2016). Accordingly, domain satisfaction is a result of how specific factors in the respective life domains (e.g., living environment) are assessed by the individual. Specific features of the places people live would not affect SWB directly, but rather indirectly through individuals’ perceptions of those life domains as a whole (e.g., Sirgy & Cornwell 2002). This intermediate step (of domain satisfaction) is derived from the assumption that individuals’ overall SWB is unlikely affected by a single factor, such as bad water quality. If only this one environmental factor is bad but others, such as friendly neighbours, cancel it out, its impact on overall SWB is likely to be small. However, if domain satisfaction (e.g., living conditions) is low due to many negative factors or a few dominant negative factors, it is assumed to impact overall SWB. According to this model, in order to understand how living conditions affect well-being, it is important not to narrow the focus to only one or two indicators, but to consider a broader range of indicators to examine combined effects or interactions (Moser, 2009).

*Setpoint or homeostasis models* build on the well documented finding that SWB and life satisfaction remain relatively level over the life cycle (e.g., Headey, 2008). According to these models, SWB fluctuates around an individual’s determined baseline level (e.g., Brickman & Campbell, 1971). The theory of SWB *homeostasis* (Cummins, 2010) assumes that mood influences SWB and mood is maintained within a (set-point) range by a homeostatic process such as cognitive reframing. However, these mechanisms can fail to protect SWB. This can occur when a certain threshold, where tolerance of objective stressors (e.g., pollution or density) was still possible, is exceeded.

Stress theory offers an explanation of why this equilibrium can be destabilized by urban living conditions. In his *stress model*, Pacione (2003) assumes that experience of the urban environment is not based on the evaluation of objective living conditions (pollution, etc.) alone, but is rather a joint function of these objective conditions and an individual evaluation based on characteristics of the person, including adaption level, previous experiences, and time spent in the city. If the environment is perceived negatively – for example, due to too many stressors – individuals experience stress and this affects their SWB. The individual may also develop coping strategies that, if successful, lead to adaption or habituation. A similar concept is proposed by Rishi and colleagues (2012). Living in a city is assumed to lead to an interaction between the adverse physical setting, due to, for example, pollution or crowding, and individual characteristics arising from the time spent exposed to these. After a certain threshold or ‘tolerance level’ is surpassed, individuals experience stress. One possible coping strategy to deal with stressors is, for example, spending time in nature. When green spaces or other positive living conditions to recover are insufficient, coping might fail but also make the described homeostatic processes – such as cognitive reframing – less successful in protecting SWB. Therefore, positive factors, such as green space, are expected to reduce the negative effects of other aspects of living conditions.

Despite some differences, these models share the assumption that differences in objective living conditions do not necessarily lead to corresponding differences in well-being. Consequently, it can be expected that objective indicators will be less strongly associated with SWB, and that the perception of living conditions is more essential. In order to reveal their relative effects, combined effects or interactions, both sources of information, objective indicators and measures of perceived living conditions, should be considered.

## Previous Research on Living Conditions and SWB

Of special interest for our research are studies directly analysing the effects of living conditions on SWB. However, physical health, mental well-being, happiness, and life satisfaction are all related and living conditions affecting physical health may also be relevant for mental health and subjective well-being. We thus include these outcomes in this overview.

### Objective and Subjective Measures of Living Conditions

A large variety of factors affect individuals’ well-being in cities, ranging from environmental problems to public transport, and so on (Aretz et al., 2019; Ma et al., 2018). A basic distinction is the use of either objective (e.g., Brereton et al., 2008) or subjective assessments of living conditions (Leyden et al., 2011; Ma et al., 2018).

Objective assessments are conducted by ‘experts’ and involve certain measures specific to the qualities of the living condition under consideration. Subjective assessments rely on self-reporting tools which enable individuals to express their evaluations of living conditions. Both measures can be reliable, valid, and useful, and thus have a high degree of measurement objectivity. At the same time, both measures contain subjectivity, considering that the more technical ‘objective’ assessments also rely on human decisions, e.g., concerning the choice of parameters (Moser, 2009). Nevertheless, for the purposes of this study, the chosen phrase ‘objective’, in contrast to our ‘subjective’ indicators, is sufficient as it is based on an external assessment of living conditions – i.e., an assessment not conducted by those subject to the living conditions in question.

Only a few studies combine both kinds of measures. Cummins (2000) found them to be fairly independent, with their dependency only increasing when objective conditions of living are very poor. Studies combining subjective measures with objective Geographic Information Systems (GIS) data in the US (Marans, 2004), and with a similar approach in Australia (McCrea et al., 2006), found rather weak links between them. Studies comparing both types of indicators repeatedly show that subjective measures predict much more of the variance in SWB than objective measures (e.g., Brereton et al., 2008; Ettema & Schekkerman, 2016; Sirgy & Cornwell, 2002). Some authors argue that these measures constitute fundamentally different concepts with different meanings as respondents’ subjective assessments are not only fed by their estimation of factual conditions, but rather by how they evaluate the impact of these conditions on their lives (Ettema & Schekkerman, 2016). This refers to the methodological problem that their impact is already inherent in their assessment of their overall SWB. The subjective assessment of living conditions is dependent on people’s preferences and values and is thus related to their assessment of their overall well-being. Several researchers therefore highlight the importance of including both types of measures, whenever possible, to create a reliable and complete picture of how living conditions impact SWB (McCrea et al., 2006; Pacione, 2003; Weckroth et al., 2022).

Two objective indicators of particular importance to this study, in addition to Mercer’s assessment of living conditions described above, are the size of cities and the level of development, as urbanisation is rapidly increasing worldwide and the number and size of megacities is growing, especially in low- and lower-middle-income countries (Andrade et al., 2012; United Nations, 2019). However, most previous research on living conditions and their impact on SWB concentrates on the developed world, mainly the US or Europe, where people report higher life evaluations than in areas with a lower Human Development Index (HDI) (Hall, 2013). The effect of city size on SWB is difficult to predict. On the one hand, (larger) cities may provide better health care, employment opportunities, education, living standards, cultural and social offers and other commodities contributing to better health and well-being among urban residents (Galea et al., 2005). On the other hand, residents of (larger) cities may be exposed to risk factors, such as overcrowding, housing issues, crime, noise and air pollution (Peen et al., 2010).

### Single Versus Multiple Indicators

The literature has considered a wide variety of living conditions that impact SWB. While most studies have focused on single indicators of living conditions, there is a lower but growing number of studies that include multiple indicators.

The immediate living environment, such as the quality and level of maintenance of housing, as well as the appearance and level of maintenance of the neighbourhood has often been analysed. The direct living environment has been found to be strongly linked to people’s overall well-being (e.g., Ma et al., 2018; Ettema & Schekkerman, 2016; Weckroth et al., 2022), and to even have an impact on physical health (Aretz et al., 2019). Based on the ‘bottom-up perspective’ (Campbell et al., 1976), the domains-of-life literature (e.g., Cummins, 1996) assumes overall life satisfaction is the result of satisfaction in several life domains. There is a consensus among many researchers on seven life domains that are of particular importance for life satisfaction and are thus used by many researchers in the same or similar form (e.g., Cummins, 1996; van Praag et al., 2003; Rojas, 2007). Besides material well-being, health, productivity, intimacy, safety, and emotional well-being, there is also a domain for ‘community’. This domain can also be referred to as ‘environment’ and generally includes factors describing the immediate living environment, often including housing and neighbourhood quality, but also factors such as pollution. Research shows that the community/environment domain plays a central role for overall life satisfaction, although it is often among the least important in this multitude of life domains (e.g., Sirgy et al., 2010; Hsieh, 2005).

Next, connectedness with the neighbourhood and social ties among residents play an important role for SWB (e.g., Ma et al., 2018; Balducci & Checchi, 2009;). Related to this are the factors crime, feeling of safety, and general neighbourhood problems, which negatively impact mental health and SWB (Dittmann & Goebel, 2010; Mair et al., 2008). When only ‘feeling safe in the neighbourhood’ is considered, it is significantly related to SWB; no such effect is found when non-safety related measures are included (Balducci & Checchi, 2009; Leyden et al., 2011; Ma et al., 2018).

Studies on environmental pollution capture ‘air pollution’, ‘water quality’ and other factors like access to sewage systems or garbage disposal. Air pollution reduces people’s subjective well-being to a significant degree (e.g., Li et al., 2018; Levinson, 2020) and at the same time has measurable effects on health and mortality (e.g., European Environment Agency, 2020). Surprisingly, none of these factors showed significant effects in any of the studies including multiple indicators (Gandelman et al., 2012; Leyden et al., 2011; Ettema & Schekkerman, 2016), except for the study by Türksever and Atalik (2001). However, these factors are often combined together or with other factors such as ‘green space’, which may distort the results.

Another indicator is ‘noise’ which, together with ‘crowding or density’, is often considered a typical negative feature of urban living conditions. Noise pollution was indeed found to negatively affect all general aspects of well-being, physical health, mental health, as well as SWB (e.g., Gandelman et al., 2012; Hammersen et al., 2016; Vienneau et al., 2015). For density, however, some studies find a clear negative effect on SWB and mental health (Guite et al., 2006; Türksever & Atalik, 2001), and sometimes there is no effect or even a reversed one (Brereton et al., 2008; Clark et al., 2006; Ma et al., 2018). One explanation could be that places with high density share characteristics such as good public transport; factors which can cancel each other out. Studies find that commuting time and transport problems are negatively and traffic safety positively associated with SWB (Ettema & Schekkerman, 2016; Ma et al., 2018; Türksever & Atalik, 2001).

Another factor which has often been studied is ‘green space’ or the quality of, and access to, nature. Green space may have direct positive effects or offset the negative effects of factors like pollution. Some studies are inconclusive, which is partly due to the strong variety in measures as well as a lack of consensus on what is the key to beneficial effects, like extent of, distance to, exposure to or the specific use of green areas (e.g., Bowler et al., 2010; Lee & Maheswaran, 2011). Nonetheless, more recent reviews and studies show associations of SWB with different green space characteristics (Hadavi, 2017; Houlden et al., 2018) as well as beneficial effects on health and mortality (Mears et al., 2020). However, except for one study suggesting an indirect effect (Gandelman et al., 2012), there is no evidence in studies with multiple indicators that greener neighbourhoods are positive for SWB or mental health (Ettema & Schekkerman, 2016; Leyden et al., 2011).

Some living conditions have been studied less often than others, such as possibilities for leisure activities (e.g., Lloyd & Auld, 2002), corruption, inner stability (e.g., Leyden et al., 2011) and health care (Alcázar & Andrade, 2010). Although all of these factors were associated with SWB, most of these studies only identify single effects of living conditions and could thus be overestimating their impact.

In sum, there is robust evidence showing that a number of indicators of living conditions impact SWB. However, when a multi-indicator approach is used, even well-established single factors may not necessarily persist in being statistically relevant.

The main aim of this study is therefore to investigate the relative importance of diverse aspects and measures of urban living conditions for SWB. To gain a comprehensive picture of the relationship between urban living conditions and SWB, we will introduce further study aims based on the presented theoretical considerations and previous empirical research. We thus investigate whether subjective factors have a stronger effect than objective factors, as found in previous research. Furthermore, we analyse whether relevant objective criteria hold additional information for explaining SWB. In additional analyses, we examine whether city size and the HDI of a country have an impact on SWB, and whether this impact is independent of subjective factors.

## Methods

### Participants and Procedure

In 2019, employees of the German Foreign Service (GFS) were invited to participate in a cross-sectional online survey. All Foreign Service employees were approached, regardless of their career path or current location. Foreign Service employees are seconded Foreign Office staff with German nationality who are posted abroad as part of their rotation. Personnel who are permanently employed in a particular location solely on the basis of employment contracts under local law and who often have the nationality of the host country were not included in the study, as this would have been beyond the scope of the study. A total of 2,250 employees participated, which corresponds to a response rate of 29.2%.

First, the heads of administration of all missions abroad and the heads of Foreign Office departments in Germany were informed about the content of the survey and asked to announce the survey to the employees. One week later, all employees were sent an email with the study invitation, the data protection information and the participation link to their personal office mailbox. Two weeks later, a reminder email was sent. The survey window was open for a total of four weeks.

Foreign Service employees are a group who face intense mobility requirements during their entire work career. Every three or four years they are assigned to new postings worldwide, usually linked to a different job with a new specification. With a few exceptions, the rotational system applies to personnel of all career paths. Participants responded anonymously, and the survey instrument and study were designed and implemented in consultation with the data protection officer and the staff council of the GFS.

Participants were 49.6% women and 50.4% men. For data protection, age was only measured in categories. The age distribution of the participants in this study corresponds almost perfectly to the distribution within the Federal Foreign Office (personnel statistics from 2019)—11% age 29 or younger, 22% 30–39, 23% 40–49, 33% 50–59, and 12% age 60 or older. Moreover, the sample shows considerably heterogeneity in terms of civil service grade (hierarchical level) based on educational level (see Table 1), with 40% of the sample belonging to the ordinary/intermediate civil servants or secretarial pool (representing the lower grades). For data protection reasons, the current location was only queried and recorded by name if there were regularly at least 25 expatriate employees there. Locations with fewer than 25 expatriate employees were grouped together. Of the about 230 diplomatic missions the German Federal Foreign Office has around the world which could be part of our sample, we are able to specify 53 cities by name (see Supplementary Information (SI), Table S8). Although we could not record the precise number of inhabitants for all locations, we could record whether it is a megacity (10 million or more inhabitants) or not.

After excluding 654 employees located in Germany and removing 377 respondents with missing information on central variables, we retain an analysis sample of 1,219. For 816 individuals, the city in which they reside is known by name, and so is the HDI and Mercer’s objective measures of living conditions. When other central variables are examined in addition to the city-level information, the sample is further reduced to 652 due to missing cases.

### Measures

#### Subjective Well-Being

Subjective well-being (SWB) is operationalized through a single item measure of overall life satisfaction. Respondents were asked: “How would you assess your total quality of life during the past weeks?” on a scale from one to seven. Self-reported overall life satisfaction is, according to Diener and Chan (2011), ‘the best single method of assessment’. Methodological studies support the validity and reliability of single-item SWB measures, including strong correlations with multi-item measures (Diener et al., 2012). The average overall SWB of the sample is 5.01 (see Table 1).

#### Subjectively Assessed Living Conditions

In the survey, 31 items representing various aspects of living conditions at the current location (i.e., city) were rated according to personal judgement. The response scale ranged from “0 - very bad” to “10 - very good”. This information was condensed into 11 dimensions using exploratory factor analysis (see SI, Table S4): ‘safety’, ‘quality of and access to nature’, ‘noise’, ‘housing’, ‘contact’, ‘congestion’, ‘quality of water, air, food supply, and sewage system’, ‘public transport/transit’, ‘accessibility of airport’, ‘sports opportunities’, and ‘medical care’. The last four dimensions each consist of a single item that could not be assigned to other dimensions. Mean value indices that consist of several individual items were formed for the dimensions (reliabilities were high, see SI, Table S4). The dimensions contain values between 0 and 10. Higher values indicate better subjective living conditions in the corresponding dimension.

#### Objectively Assessed Living Conditions

##### Mercer

In addition to subjective living conditions, objective criteria according to the international consulting firm Mercer are used for in this study. Mercer assesses quality of life by using 39 individual criteria, covering various social, political, economic and environmental aspects, and which are seen to play a central role for the posted employees (see Okulicz-Kozaryn, 2013 for full list). The criteria are rated on a scale of 0–10, with high scores expressing good living conditions in the respective aspect.

In the context of this study, 20 individual criteria were available for all named locations (the values are from September 2017). For 14 of these 20 criteria there are correspondences in the individual aspects of the subjective assessment of living conditions by the respondents at the location. In those areas where both subjective and objective indicators are available, the information provided by respondents can be compared with Mercer’s values.^3^

##### City Size

Another objective indicator is city size. A distinction was made between megacities (those with more than 10 million inhabitants) and non-megacities (United Nations, 2019). In line with previous research, we found no linear relationship between city size and SWB (Okulicz-Kozaryn & Valente, 2019 for Europe) and did not further pursue this approach.

##### Human Development Index (HDI)

To determine the general standard of living at the various locations, the Human Development Index (HDI) of the respective country was used as another objective indicator. In order to determine the HDI of a country, the UN uses four indicators that reflect the living conditions of the population across key aspects (United Nations Development Programme, 2019). These are: life expectancy at birth, expected years of education, average years of education and average gross national income per capita; these are combined into a single continuous variable, potentially ranging from 0 to 1. The lowest HDI in our sample is 0.463 (see Table 1).

#### Control Variables

In robustness checks we control for the demographic characteristics age, sex, civil service grade, partnership status, and presence of children, as well as the two dimensions of the ‘Big Five’ personality traits ‘neuroticism’ and ‘extraversion’, with complete demographic information available for 1068 individuals (see Table 1). However, as none of these control variables had a substantial impact on our main interest, and to avoid undue complexity and maximize sample size, they are not included in the main analyses (see SI, Table S5-S7).

**Table 1:** Sample Characteristics

Variables		N	Mean/%	SD	Min	Max
*Subjective well-being*		1219	5.012	1.315	1	7
*Subjective living conditions*		1219				
safety			5.953	2.266	0	10
contact			5.666	2.533	0	10
medical care			6.071	2.706	0	10
quality of and access to nature			5.008	2.958	0	10
quality of water, air, food supply, sewage system			5.000	2.671	0	10
public transport/transit			4.272	3.463	0	10
congestion			4.465	2.263	0	10
noise			5.093	2.647	0	10
accessibility of airport			6.191	2.717	0	10
sports opportunities			6.444	2.658	0	10
housing			6.080	1.982	0	10
*Objective living conditions*						
*Mercer*		816				
	inner stability		5.836	2.506	1	5.836
	crime		5.354	2.113	1	5.354
	public order, police		5.980	2.278	2	5.980
	possibilities to communicate		4.335	2.237	0	4.335
	hospitals		7.395	2.238	2	7.395
	quality of water		5.825	3.500	0	5.825
	waste disposal		6.690	1.942	1	6.690
	sewage system		7.237	2.005	3	7.237
	air pollution		4.543	2.072	1	4.543
	public transport		6.857	2.267	2	6.857
	traffic congestions		4.235	1.548	1	4.235
	sports opportunities and clubs		7.645	1.891	2	7.645
	selection and quality of housing		7.362	1.517	3	7.362
	housing maintenance and repair		7.156	2.437	1	7.156
*HDI*		816				
			0.805	0.111	0.463	0.944
*City size*		1219				
	megacity		35%	-	-	-
	non-megacity		65%	-	-	-
C*ontrol variables*		1068				
*gender*						
	female		53%	-	-	
	male		47%	-	-	-
*age group*						
	< 40		30%	-	-	-
	40–49		24%	-	-	-
	50–59		34%	-	-	-
	60 + years old		12%	-	-	-
*civil service grade (hierarchical level)*						
	secretarial pool		14%	-	-	-
	ordinary/intermediate		26%	-	-	-
	higher intermediate		39%	-	-	-
	higher		21%	-	-	-
*family status*						
	partner + no children		50%	-	-	-
	no partner + min. 1 child		4%	-	-	-
	single		15%	-	-	-
	partner + min. 1 child		31%	-	-	-
*Big 5 personality dimensions*						
	neuroticism		39.006	20.340	0	100
	extraversion		66.213	16.425	10	100

### Analyses

#### Correlation Analysis and Regression Models

We estimated zero-order correlations of each environmental living conditions variable with SWB, as well as multiple OLS regression models including the full set of subjectively and/or objectively assessed living conditions. The living conditions items assessed in our study are intercorrelated and likely have a non-simple structure related to underlying latent factors. Accordingly, to minimize the potential adverse effects of a misspecified measurement error model (Rhemtulla et al., 2020), we opted to conduct our analyses using sum scores for the living conditions scales, rather than fitting a simple-structured measurement error model. Sum scores tend to be more robust, as they have more information theoretic convergence with a wider range of possible true measurement models (cf. Burnham & Anderson, 2002). We fit additional models including city size and HDI as independent variables.^4^

#### Dominance Analysis

To compare the *relative* impacts of each environmental living conditions variable, we used dominance analysis. In a multiple regression model, when variables are correlated, the estimated regression coefficients cannot be directly interpreted as reflecting the contributions of individual variables (Budescu, 1993). Dominance analysis is a method that can allow for such comparisons. In a dominance analysis, models with all possible subsets of predictors are fit (i.e., all possible models with 1 predictor, all possible models with 2 predictors, all possible models with 3 predictors, etc.), and the patterns of increases in *R*^2^ when adding specific predictors to models is examined (Azen & Budescu, 2003). The general dominance weight for a predictor is computed as the average increase in *R*^2^ when this predictor is added to any smaller model. The general dominance weights can be interpreted as the proportion of the variance in the dependent variable that can be uniquely accounted for by the predictor (Azen & Budescu, 2003).

## Results

First, we will make a comparison of subjective and objective living conditions. Second, we will focus on subjectively assessed aspects of living conditions and their relative importance in explaining SWB.

### Comparing Objective and Subjective Measures of Living Conditions

#### Objective Measures of Living Conditions from Mercer

In our data, we have 31 single items of subjectively assessed environmental and living conditions and 20 objective criteria from Mercer. For 14 of these 20 criteria, there are equivalents in the individual aspects of the subjectively assessed factors. Therefore, in order to compare them, only these are considered in the following correlation and regression analyses.

The correlations between the 14 Mercer values and the subjective ratings are quite high: for most aspects above *r =* .50 (see Supplementary Information (SI), Table S1, third column). In other words, there is a good overall consistency of what is rated positive and what is rated negative: the better Mercer assesses the living conditions, the better the GFS employees tend to assess the same conditions. However, the correlation is much weaker with regard to some aspects, namely traffic congestion, sports opportunities, as well as maintenance and housing.

Second, we compare the influence of (single) subjective indicators on SWB with their objective Mercer equivalent to see if there is additional information coming from the objective indicators. The results show that correlations between subjective factors and SWB range between *r =* .29 and *r =* .49, whereas correlations between Mercer factors and SWB range between *r =* .10 and *r =* .29 (see SI, Table S1, first and second column). Moreover, the effects of subjective indicators are always stronger and all of them tend to lose only a little of their effect strength and remain statistically significant once objective Mercer indicators are controlled for (see regression models in SI, Table S1).

When we take into account the perspective of top-down models as well as the critique with regard to subjective factors, and only use the objective Mercer items which are completely independent of the measure of SWB, we can show that a substantial share of 11% of the variance in SWB can still be explained (R^2^ from OLS model with all 14 Mercer items: 0.11; see SI, Table S2, Model 2). When the control variables are included, the results reported in this subsection remain robust (see SI, Table S5 and Table S6, Model 2).

#### City Size and HDI

In a next step we examine if city size and HDI have effects on SWB. ‘Living in a megacity’, as an objective indicator, shows a small negative effect (*B = −* 0.23, 95% CI − 0.38, −0.08, *p =* .003) on SWB (see SI, Table S2, Model 3). However, when controlling for the subjective living condition factors using OLS regression, the effect of ‘living in a megacity’ (*B* = 0.01, 95% CI − 0.14, 0.16, *p =* .856) disappears (see SI, Table S2, Model 4), indicating that these living conditions can account for the effect of megacities on SWB.

Additional analyses (not shown) demonstrate that some specific subjectively evaluated living conditions factors – ‘quality of and access to nature’, ‘quality of water, air, food supply, and sewage system’, ‘noise’ and ‘accessibility to the airport’ – could by themselves account for the megacities impact on SWB. Including a combination of either the items ‘congestion’ and ‘safety’ or ‘congestion’ and ‘medical care’ also accounts for the negative relationship between living in a megacity and SWB. This indicates that these living conditions are crucial for the worse SWB in megacities.

The country’s HDI is another objective factor that is significantly associated with expatriates’ SWB (*B = 3*.76; 95% CI 2.96, 4.55, *p ≤* .001), but this effect also disappears when we control for subjectively evaluated living conditions (*B = −* 0.18, 95% CI − 1.20, 0.85, *p =* .735) (see SI, Table S2, Model 5 and 6). In particular, it is ‘quality of and access to nature’ and ‘quality of water, air, food supply, and sewage system’ that explain the negative effect of HDI on SWB (analysis not shown). All results for city size and HDI remain robust even when we include control variables (see SI, Table S6, Model 3–6).

Combining these results with those from the previous section on Mercer items, we see that objective indicators have substantially weaker associations with SWB, and that the perception of living conditions are more significant. We thus focus on subjectively assessed aspects of living conditions and their relative importance in explaining SWB in the following section.

### Relative Importance of Subjectively Assessed Aspects of Living Conditions

Table 2 shows the correlations, a multiple regression and a dominance analysis for the 11 subjectively evaluated dimensions of living conditions with SWB.

**Table 2:** Correlations, Regression and Dominance Analyses for Subjective Living Conditions Predicting Subjective Well-Being

Subjective living conditions		*r*	*B*	*B SE*		*β*	*β SE*	*p*	DW	%
	safety	0.380	0.019	0.019		0.033	0.033	0.327	0.022	0.063
	contact	0.324	0.045	0.015		0.086	0.028	0.002	0.023	0.066
	medical care	0.365	0.015	0.016		0.030	0.033	0.359	0.022	0.063
	quality of and access to nature	0.499	0.084	0.018		0.188	0.041	< 0.001	0.057	0.163
	quality of water, air, food supply, sewage system	0.486	0.041	0.022		0.083	0.044	0.057	0.045	0.128
	public transport/transit	0.324	-0.023	0.013		-0.061	0.034	0.069	0.014	0.041
	congestion	0.415	0.027	0.020		0.046	0.035	0.185	0.032	0.090
	noise	0.408	0.054	0.016		0.110	0.032	0.001	0.036	0.104
	accessibility of airport	0.296	0.016	0.013		0.033	0.028	0.234	0.013	0.036
	sports opportunities	0.403	0.030	0.015		0.062	0.031	0.047	0.031	0.090
	housing	0.433	0.131	0.019		0.198	0.029	< 0.001	0.055	0.157
	N (degrees of freedom)		1219 (11)							
	R^2^		0.350							
	Adj. R^2^		0.344							

*Note. r* = zero-order correlation; B = unstandardized regression coefficient; B SE = standard error for unstandardized regression coefficient; *β* = standardized regression coefficient; *β* SE = standard error for standardized regression coefficient; DW = general dominance weights (Azen & Budescu, 2003); % = percent of accounted-for SWB variance attributable to aspect of living conditions (rescaled general dominance weights). 95% CIs for *r* ± .04; 95% CIs for *β* ± 0.05; 95% CIs for *B* ± 0.04.

The bivariate correlations between the subjective factors and SWB range between *r =* .30 and *r =* .50 and are all statistically significant. The strongest correlation with SWB can be observed for the factors ‘quality of and access to nature’ (*r =* .50, 95% CI 0.46, 0.54, *p ≤* .001), ‘quality of water, air, food supply, sewage system’ (*r =* .49, 95% CI 0.45, 0.53, *p ≤* .001) and ‘housing’ (*r =* .43, 95% CI 0.39, 0.47, *p ≤* .001). The correlations for ‘congestion’ (*r =* .42, 95% CI 0.38, 0.46, *p ≤* .001), ‘noise’ (*r =* .41, 95% CI 0.37, 0.45, *p ≤* .001) and ‘sports opportunities’ (*r =* .40, 95% CI .36, .44, *p ≤* .001) are somewhat lower, but still strong. The relatively weakest correlation can be found for ‘accessibility of airport’ (*r =* .30, 95% CI. 26, .34, *p ≤* .001).

When all 11 factors are included in a multiple regression, the factors ‘medical care’, ‘safety’, ‘accessibility of airport’, and ‘congestion’ are no longer statistically significant, suggesting overlap in their impacts on SWB. The strongest effects can be found for ‘housing’ and ‘quality of and access to nature’. An increase of one unit in ‘housing’ (scale of 0–10) leads to an increase of *B* = 0.13 (95% CI 0.09, 0.17, *p* ≤ .001) units in SWB (scale of 1–7). For ‘quality of and access to nature’ the *B*-coefficient is 0.08 (95% CI 0.05, 0.12, *p* ≤ .001), to be followed by ‘noise’ (*B* = 0.05, 95% CI 0.02, 0.09, *p =* .001), ‘contact’ (*B* = 0.04, 95% CI 0.02, 0.07, *p =* .002) and ‘quality of water, air, food supply, and sewage system’ (*B* = 0.04, 95% CI − 0.00, 0.08, *p =* .057).

The subjective factors as a set correlated *R =* .59 with SWB and accounted for 35% of the variance in SWB (*R*^2^ = 0.35). Using dominance analysis, we can tell how much the different factors contribute to the accounted-for SWB variance. ‘Quality of and access to nature’ (rescaled general dominance weight = 16.3%) and ‘housing’ (15.7%) contribute most to the prediction, followed by ‘quality of water, air, food supply, and sewage system’ (12.8%) and ‘noise’ (10.4%). ‘Sports opportunities’ and ‘congestion’ account for 9% of the variance in SWB, respectively. The other factors are less important and account for only about 5% of the variance in SWB, respectively. While the effect of ‘contact’ was comparatively strong in the regression analysis, this factor contributes only 6.6% to the prediction in the dominance analysis. Moreover, ‘housing’ with the strongest effect in the regression analysis falls back behind ‘quality of and access to nature’ in its contribution to SWB in the dominance analysis. In sum, with some deviations, the results of the dominance analysis largely confirm the results of the regression analysis.

Our results show that ‘quality and access to nature’ is the most important living conditions factor associated with SWB. This is also one of the central living conditions discussed in the literature that may theoretically offset or mitigate negative living conditions. In an additional analysis, we thus examined whether this factor reduces the negative effects of any of the other ten aspects of environmental and living conditions using interaction effects (see SI, Table S3). We find that the interaction between ‘nature’ and ‘noise’ is significant, although none of the other interaction effects is. This is an interesting result as it suggests that the negative effects of noise pollution can – at least to some extent – be mitigated by higher quality and better access to nature in a city. The results also remain robust when we include control variables (see SI, Table S7).

## Discussion and Conclusion

In an increasingly urbanized world, understanding how this environment determines well-being is growing in importance. While there is a growing literature on this subject, little is known about the relevance of the various living conditions in relation to each other. The main aim of this research is therefore to examine the effect and relative importance of various urban living conditions for the subjective well-being (SWB) of German Foreign Service (GFS) expatriates. While previous research has usually investigated the effects of living conditions on residents of a certain area or city, we captured the living conditions of metropolises all over the globe and across different levels of development, taking advantage of the fact that the German Federal Foreign Office has about 230 diplomatic missions. Additionally, as the number of international assignments increases due to globalisation, it is of particular interest to investigate how the urban environment affects the well-being of expatriate city dwellers. This has not yet been studied for this group.

The empirical analyses are based on a unique dataset which provides a number of advantages, thus enriching previous research. First, it enables us to examine the relative impact of a large variety of different subjective and objective indicators of living conditions. Second, we capture the living conditions of GFS expatriates – a relatively culturally homogeneous group – in metropolises all over the globe. Our data are thus less biased by selection of place and by (statistically uncontrollable) cultural differences. Third, it provides us with the opportunity to analyse whether living in a megacity or in a developing country has an (additional) impact on SWB. This is particularly interesting as the number of megacities is growing, most of them now located in developing countries.

With the onset of the COVID-19 pandemic in early 2020, the important role that living conditions play for SWB may have become even more pronounced. Some of the environmental and other living conditions analysed in this study – particularly the availability of green spaces – are linked to the ability to maintain a physically active lifestyle with a lower risk of becoming infected, especially in densely populated places (Klompmaker et al., 2021), which may also heighten people’s awareness of the benefits of a positive living environment on physical and mental well-being.

The first important result is that both types of indicators are related, but the effects of subjective indicators are always stronger than those of their objective counterparts, supporting the results of previous research. Interestingly, despite the much smaller effects of objectively measured indicators, they also have effects beyond the (equivalent) perceived living conditions on SWB, mostly with factors concerning safety and housing quality, thus highlighting their relevance.

In our main analysis, we examine the effect and relative importance of various subjective urban living conditions for SWB. We find ‘quality of and access to nature’ (i.e., green space) to have the highest relevance for SWB, which is in line with many studies focusing solely on green space and its beneficial effects for various mental and physical well-being outcomes. It is assumed that green spaces work directly as noise barriers and heat protection, but might also reduce stress levels through providing exercise and meeting places, counterbalancing the typical stressors in urban settings.

Interestingly, in an additional analysis we find that the interaction between ‘nature’ and ‘noise’ is significant, suggesting that the negative effects of noise pollution can be mitigated by higher quality and better access to nature in a city. These results support our previously described theoretical considerations and show that we can confirm common findings from the literature with our sample of comparatively privileged expatriates, which argues against sample-specific findings. First, it supports the assumptions of the bottom-up model of SWB, which suggests that it is necessary to focus on a broader range of indicators to understand how these affect well-being, as this enables us to examine combined effects or interaction effects between different (positive and negative) living conditions. Yet, previous studies also including a variety of other living conditions found no positive effects for ‘nature’ indicators (e.g., Leyden et al., 2011; Ettema & Schekkerman, 2016). Second, it supports the assumptions of homeostasis and the stress model as the lack of green space might make it more difficult for coping strategies to be successful. It is reasonable to assume that this mitigating effect also applies, or perhaps even more so, to other, less privileged urban dwellers who may have poorer access to nature.

The second most relevant living condition is ‘quality of housing’. Despite being in line with results of previous studies, this is nevertheless a remarkable result. We expected ‘housing’ to have less variance due to the attention given and effort made by the GFS to provide quality housing irrespective of the country’s level of development. This factor might therefore be even more important for less advantaged expatriate groups or local residents. The third most important living condition is the quality of public goods (i.e., water, air, and sewage system), with the single factor ‘air pollution’ already found in previous studies to have a particularly strong impact on SWB.

We find that some living conditions – such as safety concerns, congestion, and quality of public transport – are no longer statistically significantly associated with SWB anymore when combined with other living condition. This is also in line with the theoretical assumptions described above.

We also looked into whether living in a particular urban environment has an impact on the relationship between subjectively assessed living conditions and SWB. Both living in a megacity and a lower development status of the country had negative effects on SWB. When controlling for the various indicators of living conditions – especially quality of and access to nature and public goods – the bare effect of megacities and development status vanishes. This means that their impact does not show independently, but through more negative subjective perceptions of the living conditions in those places, and that this is what explains compromised well-being.

Our study has some limitations. One of these is the cross-sectional design, obviously making it impossible to determine causal direction, meaning, for example, that happier people might evaluate their environment more positively. This is a common issue in research on the determinants of SWB which cannot be ruled out in cross-sectional designs.

Another problem is that different subjective assessments are generally more strongly correlated with each other than with objective indicators. This may be due at least in part to the fact that there is a common person factor that is reflected in both assessment processes, in the subjective assessment of one’s external living conditions and in the subjective assessment of one’s own well-being, whereas no person factor is inherent in the objective indicators.

It has to be left to future research, e.g., with longitudinal design, to verify whether the living conditions we found to be associated with SWB also show a similar impact when unobserved heterogeneity is controlled for. Previous research suggests including objective indicators, instead of focusing on perceived living conditions, provides a more reliable picture as a causal relationship can then be established. However, objective assessment might only reflect the general situation in the respective cities and therefore has less to do with the respective life reality on site, most likely explaining the weak effect we found and also underlining the necessity of considering both types of indicators in future research.

Our sample undoubtedly represents a selective group of city dwellers. These are expatriates living in a foreign environment, having a stable income and good access to high quality living conditions. When moving to another city they can rely on a certain range of support from their employer and, in most cases, they can draw on previous experience from other assignments. This may contribute to better coping with the downsides of living in larger cities and thus to a higher well-being. However, whether and how the socio-demographic and socio-economic characteristics of Foreign Service personnel have an impact on the relationship between urban living conditions and SWB is unclear. The literature concerning the impact of these variables also does not support a conclusion. For example, on the one hand, higher income could be associated with higher sensitivity to air pollution (Cheng et al., 2022). On the other hand, people with higher incomes may be better able to protect themselves against the adverse effects of air pollution, for example through air filters (Zhan, Zhan, & Che, 2017). Overall, more research is needed on these issues.

Furthermore, the importance of living conditions for SWB might shift across life stages. However, whether our results apply beyond the group of working-age people cannot be answered with our data and must be left to future research.

Nevertheless, for several reasons, we argue that our findings concerning living conditions may be of interest beyond the group under study – (a) consistent with previous literature, ‘nature’ and ‘housing’ proved to be particularly relevant for SWB in our study, pointing to a more general relevance of the result; (b) despite its perhaps lower variance in our sample, ‘housing’ had a high explanatory value for SWB, suggesting that it might even increase in a more representative sample of urban inhabitants; (c) also consistent with the literature, ‘nature’ mitigated the negative influence of other variables such as ‘noise’. For less privileged urban dwellers with poorer access to nature, this mitigating effect is presumably also true – or perhaps even more so. However, the question to what extent the results can be generalized to the broader urban population cannot be answered with absolute certainty.

In any case, the results have implications for designing psychological and other support measures to help Foreign Service employees or other groups of internationally deployed staff to prepare for, adapt to, and respond to their new urban environments. In addition, they may have broader implications for environmental design and may also be useful to city planners and local decision makers who have the whole urban population in mind.

In sum, our results suggest that locations with low quality living conditions – in the form of lack of green spaces, low quality housing, and public goods – should be perceived as threats to SWB.

## Electronic Supplementary Material

Below is the link to the electronic supplementary material.

Supplementary Material 1

## Footnotes

1 In a survey of GFS expatriates, 31 items representing various aspects of urban environmental and living conditions were rated according to personal judgement. In preparatory analyses, this information was condensed into the 11 dimensions mentioned above using exploratory factor analysis (see Methods section). For the purposes of brevity, throughout the rest of this paper we will use the term ‘living conditions’ to refer to all dimensions under consideration.

2 We note, however, that our sample shows a remarkable heterogeneity in terms of civil service grade (hierarchical level) based on educational level (see Table 1), with 40% of the sample belonging to the ordinary/intermediate civil servants or secretarial pool (representing the lower grades).

3 It is possible that Mercer’s indicators relate more to the urban level and that the respondents’ subjective assessments relate more to their immediate living environment. However, the latter does not seem to apply to all aspects of living conditions examined. Rather, several of the subjectively assessed aspects of living conditions tend to reflect the urban level, such as public transportation, medical care, hospitals, drinking water quality, food safety, air quality, waste and sewage disposal, and general noise. In addition, it is likely that respondents often abstracted from their immediate living and working environments when evaluating living conditions in their localities and also considered the broader conditions in their respective cities. Moreover, the literature suggests that both neighbourhood (i.e., immediate living conditions) and urban characteristics are important for life satisfaction (Ettema & Schekkerman, 2016).

4 In further robustness analyses, we additionally included city fixed-effects in the regression analyses (results not shown). However, this leads to a significant and selective reduction in the number of cases. Moreover, the main results do not change. Therefore, we report the analyses without city fixed-effects. Due to data restrictions, it was not possible to use multi-level analyses.
