# Supplementary material for: The Effects of Urban Living Conditions on Subjective Well-Being: The Case of German Foreign Service Employees
Source: Appl Res Qual Life. 2023 Apr 22:1–25. Online ahead of print. doi: 10.1007/s11482-023-10169-w (PMC10122088; doi:10.1007/s11482-023-10169-w)
Supplement: Supplementary file 1 — Supplementary Material 1 [file 11482_2023_10169_MOESM1_ESM.pdf]

Supplementary Information

Heiko Rüger, Stefanie Hoherz, Norbert F. Schneider, Herbert Fliege, Maria M. Bellinger, and Brenton M. Wiernik: The Effects of Urban Living Conditions on Subjective Well-being: The Case of German Foreign Service Employees. Applied Research in Quality of Life.

Corresponding author:
Heiko Rüger
Federal Institute for Population Research (BiB)
Friedrich-Ebert-Allee 4
65185 Wiesbaden, Germany
Email: heiko.rueger@bib.bund.de

Table S1

Single aspects of subjective living conditions (LC) compared with their Mercer equivalent: Correlations, OLS regressions on SWB for each living condition

	Bivariate correlations			Linear regression models for													
				each subj. LC on SWB separately; not controlled for objective LC (Mercer) equivalent[1]					each subj. LC on SWB separately; + objective LC (Mercer) equivalent controlled for[1]								
				Subjective LC coeff.					Subjective LC coeff.			Objective LC coeff.					
Living conditions (subjective /objective indicator)[2]	subj. LC with SWB	obj. LC with SWB	subj. LC with obj. LC	B	B SE	P>|t|	R	R^2	B	B SE	P>|t|	B	B SE	P>|t|	R	R^2	N[3]
political stability/inner stability	0.302	0.257	0.624	0.139	0.016	< 0.001	0.091	0.090	0.107	0.020	< 0.001	0.055	0.021	0.010	0.099	0.097	794
crime, corruption/crime	0.342	0.257	0.571	0.144	0.014	< 0.001	0.117	0.116	0.122	0.017	< 0.001	0.052	0.024	0.032	0.123	0.120	758
public order/public order, police	0.395	0.288	0.591	0.171	0.014	< 0.001	0.156	0.155	0.149	0.017	< 0.001	0.046	0.022	0.037	0.161	0.158	797
possibilities to communicate	0.269	0.123	0.537	0.111	0.014	< 0.001	0.072	0.071	0.118	0.017	< 0.001	-0.019	0.023	0.403	0.073	0.071	805
medical care, hospitals/hospitals	0.411	0.249	0.520	0.214	0.018	< 0.001	0.169	0.168	0.201	0.021	< 0.001	0.026	0.022	0.251	0.171	0.168	719
quality of water	0.423	0.285	0.603	0.163	0.012	< 0.001	0.179	0.178	0.152	0.016	< 0.001	0.017	0.014	0.223	0.181	0.179	782
waste disposal and sewage system/waste disposal	0.420	0.286	0.598	0.160	0.012	< 0.001	0.177	0.175	0.148	0.015	< 0.001	0.035	0.026	0.181	0.178	0.176	782
waste disposal and sewage system/sewage system	0.420	0.289	0.584	0.160	0.012	< 0.001	0.177	0.175	0.146	0.015	< 0.001	0.040	0.025	0.108	0.179	0.177	782
air quality/air pollution	0.439	0.225	0.568	0.177	0.013	< 0.001	0.193	0.192	0.185	0.016	< 0.001	-0.023	0.023	0.331	0.194	0.192	803
public transport	0.356	0.260	0.704	0.130	0.012	< 0.001	0.127	0.126	0.124	0.017	< 0.001	0.011	0.026	0.662	0.127	0.125	784

(Table S1 continued)	Bivariate correlations			Linear regression models for													
				each subj. LC on SWB separately; not controlled for objective LC (Mercer) equivalent[1]					each subj. LC on SWB separately; + objective LC (Mercer) equivalent controlled for[1]								
				Subjective LC coeff.					Subjective LC coeff.			Objective LC coeff.					
Living conditions (subjective /objective indicator)[2]	subj. LC with SWB	obj. LC with SWB	subj. LC with obj. LC	B	B SE	P>|t|	R	R^2	B	B SE	P>|t|	B	B SE	P>|t|	R	R^2	N[3]
traffic congestion	0.306	0.162	0.432	0.145	0.016	< 0.001	0.094	0.092	0.138	0.018	< 0.001	0.029	0.030	0.344	0.095	0.092	802
sports opportunities/ sports opportunities and clubs	0.421	0.198	0.316	0.200	0.015	< 0.001	0.177	0.176	0.189	0.016	< 0.001	0.050	0.022	0.026	0.182	0.180	786
selection and quality of housing	0.294	0.153	0.107	0.164	0.019	< 0.001	0.087	0.085	0.156	0.019	< 0.001	0.101	0.029	< 0.001	0.101	0.099	744
housing maintenance and repair	0.360	0.252	0.103	0.170	0.016	< 0.001	0.130	0.129	0.160	0.015	< 0.001	0.112	0.017	< 0.001	0.178	0.176	781

Note. B = unstandardized regression coefficient. B SE = standard error of unstandardized regression coefficient.
All correlations between SWB and subjective and objective living conditions items are stat. significant ($p \leq 0.001$). 95% CIs for r ± .04. 95% CIs for B ± .06.

[1] Each row depicts a regression model with only either the subjective LC controlled for or the subjective LC + the equivalent objective LC (Mercer).
[2] In cases where the label varies between subjective and objective indicator, each label is listed separated by a '/'.
[3] The analyses in each line are based on the same sample, and the observation numbers are thus identical (and reported once). The degrees of freedom are always 1 in models controlling only for the subjective LC, and 2 for models including the subjective and the objective LC.

Table S2

OLS Regression for SWB with 11 dimensions of subjective living conditions, with all 14 Mercer living condition items that have single item of subjective living conditions equivalents, Megacity, and HDI

	Model 1			Model 2			Model 3			Model 4			Model 5			Model 6		
Variable	B	B SE	P>|t|	B	B SE	P>|t|	B	B SE	P>|t|	B	B SE	P>|t|	B	B SE	P>|t|	B	B SE	P>|t|
Subjective living conditions																		
safety	0.019	0.019	0.327							0.019	0.019	0.334				0.062	0.025	0.016
contact	0.045	0.015	0.002							0.044	0.015	0.003				0.024	0.019	0.214
medical care	0.015	0.016	0.359							0.014	0.016	0.378				-0.002	0.023	0.924
quality of and access to nature	0.084	0.018	< 0.001							0.084	0.018	< 0.001				0.064	0.023	0.006
quality of water, air, food supply, sewage system	0.041	0.022	0.057							0.041	0.022	0.056				0.087	0.029	0.003
public transport/transit	-0.023	0.013	0.069							-0.024	0.013	0.072				-0.028	0.018	0.110
congestion	0.027	0.020	0.185							0.028	0.021	0.188				0.021	0.026	0.432
noise	0.054	0.016	0.001							0.055	0.016	0.001				0.036	0.021	0.088
accessibility of airport	0.016	0.013	0.234							0.016	0.014	0.230				0.021	0.017	0.220
sports opportunities	0.030	0.015	0.047							0.030	0.015	0.049				0.030	0.020	0.137
housing	0.131	0.019	< 0.001							0.131	0.019	< 0.001				0.142	0.026	< 0.001
Objective living conditions (Mercer)																		
inner stability				0.089	0.040	0.024												
crime				-0.030	0.044	0.491												
public order, police				-0.089	0.070	0.199												
possibilities to communicate				-0.017	0.025	0.483												
hospitals				0.082	0.055	0.136												
quality of water				0.039	0.034	0.247												
waste disposal				0.052	0.063	0.412												
sewage system				0.077	0.086	0.368												
air pollution				0.106	0.040	0.008												
public transport				0.123	0.041	0.003												
traffic congestions				-0.027	0.040	0.499												
sports opportunities and clubs				-0.106	0.068	0.122												
selection and quality of housing				-0.078	0.057	0.171												
housing maintenance and repair				-0.075	0.064	0.243												

(Table S2 continued)	Model 1			Model 2			Model 3			Model 4			Model 5			Model 6		
Variable	B	B SE	P>|t|	B	B SE	P>|t|	B	B SE	P>|t|	B	B SE	P>|t|	B	B SE	P>|t|	B	B SE	P>|t|
City size																		
megacity							-0.231	0.078	0.003	0.014	0.076	0.856						
(ref.: non-megacity)																		
HDI																		
(continuous)													3.755	0.407	<0.001	-0.177	0.522	0.735
N (degrees of freedom)	1219	(11)		816	(14)		1219	(1)		1219	(12)		652	(1)		652	(12)	
R^2	0.350			0.113			0.007			0.350			0.116			0.380		
Adj. R^2	0.344			0.097			0.006			0.344			0.114			0.369		

Note. B = unstandardized regression coefficient. B SE = standard error of unstandardized regression coefficient. 95% CIs for B ± .79.

Table S3

OLS Regression for SWB with 11 dimensions of subjective living conditions and 'quality of and access to nature' as moderator

	Model 1			Model 2		
Variable	B	B SE	P>|t|	B	B SE	P>|t|
Subjective living conditions						
safety	0.019	0.019	0.327	-0.024	0.036	0.499
contact	0.045	0.015	0.002	0.068	0.028	0.017
medical care	0.015	0.016	0.359	0.026	0.029	0.376
quality of and access to nature	0.084	0.018	< 0.001	0.189	0.047	< 0.001
quality of water, air, food supply, sewage system	0.041	0.022	0.057	0.068	0.041	0.103
public transport/transit	-0.023	0.013	0.069	-0.025	0.027	0.357
congestion	0.027	0.020	0.185	0.078	0.039	0.045
noise	0.054	0.016	0.001	0.089	0.026	0.001
accessibility of airport	0.016	0.013	0.234	0.046	0.024	0.060
sports opportunities	0.030	0.015	0.047	0.005	0.027	0.854
housing	0.131	0.019	< 0.001	0.143	0.035	< 0.001
Interaction with quality of and access to nature						
safety				0.011	0.007	0.113
contact				-0.004	0.005	0.474
medical care				-0.002	0.005	0.678
quality of water, air, food supply, sewage system				-0.006	0.007	0.400
public transport/transit				0.001	0.005	0.837
congestion				-0.011	0.007	0.086
noise				-0.008	0.004	0.044
accessibility of airport				-0.006	0.005	0.177
sports opportunities				0.007	0.005	0.226
housing				-0.003	0.007	0.602
N (degrees of freedom)	1219	(11)		1219	(21)	
R^2	0.350			0.363		
Adj. R^2	0.344			0.352		

Note. B = unstandardized regression coefficient. B SE = standard error of unstandardized regression coefficient. 95% CIs for B ± .09.

Table S4

Exploratory factor analysis for 31 subjectively assessed living conditions items

Variables	safety	quality of and access to nature	noise	housing	contact	congestion	quality of water, air, food supply, sewage system
offer/ selection of apartments				**0.626**			-0.264
quality of the housing estate		0.193		**0.672**			
quality of the apartment				**0.874**			
maintenance and repair services in the household	0.122			**0.658**			0.159
green spaces: presence, accessibility		**0.948**					
green spaces: quality		**0.898**					
nature: presence, accessibility, quality		**0.697**				0.159	
sports opportunities							
outdoor activities		**0.683**				0.110	0.172
medical care, hospitals		0.174	0.253		0.246	0.363	-0.141
drinking water quality	0.127	0.227		0.101	0.109		**0.551**
food safety	0.116	0.179		0.165	0.147		**0.515**
air quality		0.255	0.162			0.288	**0.373**
waste and sewage disposal	0.212	0.270					**0.384**
public transportation		0.301			-0.124	0.290	
traffic density, traffic jams	0.151	0.117	0.159		-0.194	**0.474**	0.165
flight connections, accessibility of the airport		0.252					0.148
general noise			**0.570**		-0.127	0.366	
noise in the living environment			**0.987**				
nightly noise			**0.906**				
possibilities to communicate	0.111				**0.623**		0.109
ability to learn the national language					**0.745**		
contact possibilities with locals					**0.732**	0.116	-0.107
social density, tightness					0.334	**0.654**	
congestion, queues						**0.649**	
political stability	**0.592**				0.229		0.243
reliability of public order	**0.699**	0.122			0.130		0.160
social tensions	**0.703**				0.110		0.114
burglary security at home	**0.678**			0.121	-0.110		-0.165
security in public space	**0.842**				-0.124		-0.197
other crime, corruption	**0.717**						0.146
Cronbach's α	0.880	0.873	0.886	0.887	0.895	0.882	0.871
Number of cases	2056	2152	2125	2124	1894	2007	2113

Note. Values below .1 are not shown. Items grouped together are bold faced. Four single items (sports opportunities; public transportation; medical care, hospitals; flight connections, access of the airport) that could not be grouped together into indices are in italics. Cronbach's α was calculated for indices. The factor analysis is a "pre-analysis" and is thus based on the full sample with employees located both in Germany and abroad.

Table S5

Single aspects of subjective living conditions (LC) compared with their Mercer equivalent: OLS regression on SWB for each living condition + controls

	Bivariate correlations			Linear regression models for													
				each subj. LC on SWB separately; not controlled for objective LC (Mercer) equivalent[1]					each subj. LC on SWB separately; + objective LC (Mercer) equivalent controlled for[1]								
				Subjective LC coeff.					Subjective LC coeff.			Objective LC coeff.					
Living conditions (subjective /objective indicator)[2]	subj. LC with SWB	obj. LC with SWB	subj. LC with obj. LC	B	B SE	P>|t|	R	R^2	B	B SE	P>|t|	B	B SE	P>|t|	R	R^2	N[3]
political stability/inner stability	0.319	0.261	0.651	0.145	0.017	< 0.001	0.128	0.117	0.109	0.022	< 0.001	0.060	0.023	0.066	0.149	0.127	683
crime, corruption/crime	0.323	0.259	0.585	0.147	0.015	< 0.001	0.137	0.126	0.124	0.019	< 0.001	0.056	0.026	0.147	0.174	0.153	653
public order/public order, police	0.389	0.284	0.618	0.180	0.015	< 0.001	0.180	0.170	0.163	0.019	< 0.001	0.035	0.023	0.237	0.218	0.198	685
possibilities to communicate	0.248	0.117	0.565	0.105	0.015	< 0.001	0.089	0.078	0.107	0.018	< 0.001	-0.004	0.025	0.656	0.092	0.070	692
medical care, hospitals/hospitals	0.376	0.255	0.535	0.225	0.019	< 0.001	0.170	0.160	0.217	0.023	< 0.001	0.016	0.024	0.468	0.218	0.198	625
quality of water	0.405	0.277	0.636	0.157	0.013	< 0.001	0.193	0.183	0.144	0.017	< 0.001	0.020	0.016	0.362	0.208	0.187	673
waste disposal and sewage system/waste disposal	0.392	0.325	0.644	0.165	0.013	< 0.001	0.187	0.177	0.151	0.017	< 0.001	0.038	0.028	0.201	0.228	0.208	675
waste disposal and sewage system/sewage system	0.392	0.309	0.632	0.165	0.013	< 0.001	0.187	0.177	0.150	0.016	< 0.001	0.040	0.026	0.270	0.228	0.208	675
air quality/air pollution	0.426	0.245	0.584	0.177	0.014	< 0.001	0.212	0.202	0.187	0.017	< 0.001	-0.025	0.025	0.555	0.239	0.220	690
public transport	0.337	0.267	0.730	0.139	0.013	< 0.001	0.150	0.139	0.130	0.019	< 0.001	0.017	0.029	0.832	0.176	0.155	673
traffic congestion	0.294	0.177	0.464	0.147	0.017	< 0.001	0.116	0.105	0.138	0.019	< 0.001	0.034	0.032	0.327	0.137	0.115	691
sports opportunities/ sports opportunities and clubs	0.408	0.208	0.320	0.205	0.016	< 0.001	0.197	0.188	0.191	0.017	< 0.001	0.059	0.024	0.021	0.217	0.197	675

(Table S5 continued)	Bivariate correlations			Linear regression models for													
				each subj. LC on SWB separately; not controlled for objective LC (Mercer) equivalent[1]					each subj. LC on SWB separately; + objective LC (Mercer) equivalent controlled for[1]								
				Subjective LC coeff.					Subjective LC coeff.			Objective LC coeff.					
Living conditions (subjective /objective indicator)[2]	subj. LC with SWB	obj. LC with SWB	subj. LC with obj. LC	B	B SE	P>|t|	R	R^2	B	B SE	P>|t|	B	B SE	P>|t|	R	R^2	N[3]
selection and quality of housing	0.302	0.200	0.160	0.161	0.021	< 0.001	0.118	0.107	0.150	0.021	< 0.001	0.113	0.031	0.001	0.152	0.129	658
housing maintenance and repair	0.360	0.263	0.134	0.175	0.017	< 0.001	0.161	0.151	0.162	0.017	< 0.001	0.119	0.018	0.000	0.226	0.206	671

Note. B = unstandardized regression coefficient. B SE = standard error of unstandardized regression coefficient.
All correlations between SWB and subjective and objective living conditions items are stat. significant ($p \leq 0.001$).

[1] Each row depicts a regression model with only either the subjective LC controlled for or the subjective LC + the equivalent objective LC (Mercer).
[2] In cases where the label varies between subjective and objective indicator, each label is listed separated by a '/'.
[3] The analyses in each line are based on the same sample, and the observation numbers are thus identical (and reported once). The degrees of freedom are always 13 in models controlling only for the subjective LC, and 14 for models including the subjective and the objective LC.
This table is identical to Table S1, except all 28 models include control variables: gender, age group, civil service group, family status, Big 5 personality dimensions 'extraversion' and 'neuroticism'.
The coefficients of the control variables are not included in the table for clarity.

Table S6

OLS Regression for SWB with 11 dimensions of subjective living conditions, with all Mercer living condition items that have single item of subjective living conditions equivalents, Megacity, HDI, + control variables

	Model 1			Model 2			Model 3			Model 4			Model 5			Model 6		
Variable	B	B SE	P>|t|	B	B SE	P>|t|	B	B SE	P>|t|	B	B SE	P>|t|	B	B SE	P>|t|	B	B SE	P>|t|
Subjective living conditions																		
safety	0.017	0.021	0.424							0.017	0.021	0.411				0.062	0.028	0.026
contact	0.039	0.016	0.014							0.040	0.016	0.013				0.020	0.020	0.328
medical care	0.019	0.017	0.267							0.020	0.017	0.249				0.019	0.025	0.446
quality of and access to nature	0.099	0.019	<0.001							0.098	0.019	<0,001				0.079	0.025	0.002
quality of water, air, food supply, sewage system	0.026	0.023	0.252							0.025	0.023	0.270				0.062	0.031	0.045
public transport/transit	-0.016	0.014	0.237							-0.014	0.014	0.308				-0.021	0.019	0.277
congestion	0.013	0.022	0.543							0.010	0.023	0.648				-0.002	0.028	0.942
noise	0.057	0.017	0.001							0.057	0.017	0.001				0.046	0.022	0.039
accessibility of airport	0.016	0.014	0.271							0.015	0.014	0.297				0.027	0.019	0.164
sports opportunities	0.030	0.016	0.059							0.031	0.016	0.056				0.036	0.021	0.085
housing	0.129	0.021	<0.001							0.130	0.021	<0,001				0.129	0.028	<0,000
Objective living conditions (Mercer)																		
inner stability				0.112	0.043	0.010												
crime				-0.010	0.049	0.832												
public order, police				-0.136	0.076	0.076												
possibilities to communicate				-0.010	0.026	0.713												
hospitals				0.072	0.060	0.233												
quality of water				0.019	0.036	0.593												
waste disposal				0.088	0.068	0.196												
sewage system				0.003	0.091	0.977												
air pollution				0.127	0.044	0.004												
public transport				0.136	0.045	0.002												
traffic congestions				-0.023	0.044	0.605												
sports opportunities and clubs				-0.055	0.076	0.472												
selection and quality of housing				-0.105	0.063	0.094												
housing maintenance and repair				-0.037	0.070	0.593												

(Table S6 continued)	Model 1			Model 2			Model 3			Model 4			Model 5			Model 6		
Variable	B	B SE	P>|t|	B	B SE	P>|t|	B	B SE	P>|t|	B	B SE	P>|t|	B	B SE	P>|t|	B	B SE	P>|t|
City size																		
megacity							-0.246	0.084	0.003	-0.032	0.081	0.695						
(ref.: non-megacity)																		
HDI													3.784	0.436	<0,001	-0.306	0.556	0.582
(continuous)																		
Gender																		
female	0.052	0.077	0.502	-0.006	0.104	0.957	0.157	0.095	0.099	0.052	0.077	0.502	0.006	0.118	0.957	-0.068	0.100	0.493
(ref.: male)																		
Age group																		
40-49	-0.026	0.090	0.773	-0.194	0.125	0.120	-0.046	0.111	0.680	-0.026	0.090	0.770	-0.163	0.138	0.240	-0.071	0.117	0.545
50-59	0.035	0.083	0.676	0.089	0.114	0.436	0.042	0.101	0.680	0.035	0.083	0.675	0.157	0.127	0.215	0.187	0.108	0.084
60+ years old	0.112	0.118	0.344	-0.075	0.162	0.644	0.097	0.143	0.498	0.111	0.118	0.350	0.029	0.182	0.873	0.188	0.156	0.229
(ref.: <40 years old)																		
Civil service grade																		
ordinary/intermediate	-0.063	0.118	0.596	0.170	0.165	0.304	-0.044	0.146	0.762	-0.061	0.119	0.606	0.002	0.183	0.993	-0.027	0.154	0.862
higher intermediate	-0.042	0.108	0.697	0.240	0.149	0.109	0.128	0.132	0.333	-0.041	0.108	0.707	0.145	0.164	0.378	-0.019	0.140	0.890
higher	0.110	0.128	0.390	0.426	0.169	0.012	0.391	0.156	0.012	0.114	0.128	0.372	0.297	0.188	0.115	0.056	0.161	0.730
(ref.: secretarial pool)																		
Family status																		
partner + no children	-0.099	0.078	0.207	-0.147	0.109	0.179	-.0219	0.096	0.024	-0.102	0.079	0.197	-0.115	0.119	0.333	-0.107	0.101	0.289
no partner + min. 1 child	0.050	0.181	0.781	-0.272	0.245	0.267	-0.041	0.223	0.853	0.050	0.181	0.782	0.024	0.260	0.927	-0.015	0.220	0.947
single	0.004	0.105	0.969	0.083	0.138	0.546	0.000	0.128	0.999	0.002	0.105	0.984	0.117	0.159	0.460	-0.002	0.135	0.988
(ref.: partner + min. 1 child)																		
Big 5 personality dimensions																		
Neuroticism (continuous)	-0.008	0.002	0.000	-0.006	0.002	0.008	-0.008	0.002	<0,001	-0.008	0.002	<0,001	-0.007	0.003	0.008	-0.007	0.002	0.003
Extraversion (continuous)	0.001	0.002	0.561	0.004	0.003	0.181	-0.001	0.003	0.782	0.001	0.002	0.559	0.004	0.003	0.214	0.003	0.003	0.285
N (degrees of freedom)	1068	(23)		700	(26)		1068	(13)		1068	(24)		568	(13)		568	(24)	
R^2	0.383			0.152			0.041			0.383			0.150			0.419		
Adj. R^2	0.369			0.119			0.029			0.369			0.130			0.394		

Note. B = unstandardized regression coefficient. B SE = standard error of unstandardized regression coefficient. This table is identical to Table S2, except all models include control variables.

Table S7

OLS Regression for SWB with 11 dimensions of subjective living conditions and 'quality of and access to nature' as moderator + controls

	Model 1			Model 2		
Variable	B	B SE	P>|t|	B	B SE	P>|t|
Subjective living conditions						
safety	0.017	0.021	0.424	-0.019	0.039	0.619
contact	0.039	0.016	0.014	0.081	0.030	0.007
medical care	0.019	0.017	0.267	0.020	0.031	0.551
quality of and access to nature	0.099	0.019	<0.001	0.220	0.048	0.000
quality of water, air, food supply, sewage system	0.026	0.023	0.252	0.059	0.043	0.142
public transport/transit	-0.016	0.014	0.237	-0.017	0.028	0.581
congestion	0.013	0.022	0.543	0.055	0.041	0.299
noise	0.057	0.017	0.001	0.105	0.027	0.000
accessibility of airport	0.016	0.014	0.271	0.045	0.026	0.083
sports opportunities	0.030	0.016	0.059	-0.008	0.028	0.712
housing	0.129	0.021	<0.001	0.152	0.036	0.000
Interaction with quality of and access to nature						
safety				0.010	0.007	0.207
contact				-0.008	0.005	0.133
medical care				0.000	0.006	0.960
quality of water, air, food supply, sewage system				-0.008	0.008	0.338
public transport/transit				0.001	0.005	0.916
congestion				-0.009	0.007	0.401
noise				-0.010	0.004	0.024
accessibility of airport				-0.006	0.005	0.211
sports opportunities				0.010	0.006	0.069
housing				-0.006	-0.007	0.361
Gender						
female	0.052	0.077	0.502	0.064	0.077	0.408
(ref.: male)						
Age group						
40-49	-0.026	0.090	0.773	0.016	0.089	0.857
50-59	0.035	0.083	0.676	0.082	0.083	0.322
60+ years old	0.112	0.118	0.344	0.157	0.118	0.186
(ref.: <40 years old)						
Civil service grade						
ordinary/intermediate	-0.063	0.118	0.596	-0.057	0.118	0.627
higher intermediate	-0.042	0.108	0.697	-0.068	0.108	0.529
higher	0.110	0.128	0.390	0.089	0.127	0.482
(ref.: secretarial pool)						
Family status						
partner + no children	-0.099	0.078	0.207	-0.109	0.078	0.165
no partner + min. 1 child	0.050	0.181	0.781	0.040	0.181	0.825
single	0.004	0.105	0.969	-0.003	0.105	0.977
(ref.: partner + min. 1 child)						
Big 5 personality dimensions						
Neuroticism (continuous)	-0.008	0.002	0.000	-0.009	0.002	0.000
Extraversion (continuous)	0.001	0.002	0.561	0.002	0.002	0.378
N (degrees of freedom)	1068	(23)			1068	(33)
R^2	0.383				0.401	
Adj. R^2	0.369				0.381	

Note. B = unstandardized regression coefficient. B SE = standard error of unstandardized regression coefficient.
This table is identical to Table S3, except all models include control variables.

Table S8

Overview of the cities included with name

City	Country	Megacity[b]	HDI[c]
**Africa**			
Abuja	Nigeria	No	0.532
Addis Ababa	Ethiopia	No	0.463
Algiers	Algeria	No	0.754
Cairo	Egypt	Yes	0.696
Lagos	Nigeria	Yes	0.532
Nairobi	Kenya	No	0.590
Pretoria	South Africa	No	0.699
Rabat	Morocco	No	0.667
Tunis	Tunisia	No	0.735
**Asia**			
Amman	Jordan	No	0.735
Ankara[a]	Turkey	No	0.791
Baghdad	Iraq	No	0.692
Bangkok	Thailand	Yes	0.755
Beijing	China	Yes	0.752
Beirut	Lebanon	No	0.757
Erbil	Iraq	No	0.685
Hanoi	Vietnam	No	0.694
Islamabad	Pakistan	No	0.562
Istanbul[a]	Turkey	Yes	0.791
Jakarta	Indonesia	Yes	0.694
Kabul	Afghanistan	No	0.498
New Delhi	India	Yes	0.640
Riyadh	Saudi Arabia	No	0.853
Shanghai	China	Yes	0.752
Singapore	Singapore	No	0.932
Tehran	Iran	Yes	0.798
Tel Aviv-Yafo	Israel	No	0.903
Tokyo	Japan	Yes	0.909
**Europe**			
Athens	Greece	No	0.870
Belgrade	Serbia	No	0.787
Bern	Switzerland	No	0.944
Brussels	Belgium	No	0.916
Bucharest	Romania	No	0.811
Geneva	Switzerland	No	0.944
Kyiv	Ukraine	No	0.751
London	Great Britain	Yes	0.922
Madrid	Spain	No	0.891
Minsk	Belarus	No	0.839
Moscow	Russia	Yes	0.816
Paris	France	Yes	0.901
Pristina	Kosovo	No	0.787
Rome	Italy	No	0.880
Sofia	Bulgaria	No	0.813
The Hague	Netherlands	No	0.931
Tirana	Albania	No	0.785
Vienna	Austria	No	0.908
Warsaw	Poland	No	0.865
**Latin America**			
Bogotá	Colombia	Yes	0.747
Brasília	Brazil	No	0.759
Buenos Aires	Argentina	Yes	0.825
Mexico City[a]	Mexico	Yes	0.774
**North America**			
New York City	USA	Yes	0.924
Washington, D.C.	USA	No	0.924

[a] According to the UN definition, which includes socio-cultural factors in addition to geographical location in the classification, Turkish cities are assigned to Asia. Mexico City, also following the UN, is categorized as Latin America on the basis of language and demographic factors (United Nations, 2018).

[b] For the distinction between megacities and non-megacities we follow, with a small deviation, the UN definition (2019) that classifies cities with more than 10 million inhabitants as megacities. This distinction is sometimes based only on the city's population, sometimes on the urban agglomeration, and sometimes the metropolitan area is considered. For three cities (Seoul, Tehran, and London) with just below 10 million inhabitants, we departed from the UN definition and classified them as megacities.

[c] The Human Development Index (HDI) is a summary measure of a country's average achievement in key dimensions of human development: life expectancy at birth, expected years of education, average years of education, and average gross national income per capita (United Nations Development Programme, 2018).
